# Metronidazole therapy as initial treatment of *Clostridium difficile* infection in patients with chronic kidney disease in Korea

**DOI:** 10.1017/S0950268819001742

**Published:** 2019-10-14

**Authors:** Jaeuk Shin, Yu Mi Wi, Yu-Ji Lee

**Affiliations:** 1 Division of Gastroenterology, Department of Medicine, Changwon Fatima Hospital, Changwon, Korea; 2 Division of Infection, Department of Medicine, Samsung Changwon Hospital, Sungkyunkwan University School of Medicine, Changwon, Korea; 3 Division of Nephrology, Department of Medicine, Samsung Changwon Hospital, Sungkyunkwan University School of Medicine, Changwon, Korea

**Keywords:** Chronic kidney disease, *Clostridium difficile*, dialysis, metronidazole, treatment failure

## Abstract

The risk of metronidazole treatment failure in *Clostridium difficile* infection (CDI) patients with chronic kidney disease (CKD) or end-stage renal disease in Korea has not been established. We evaluated 481 patients who had been admitted to two secondary hospitals with a diagnosis of, and treatment for, CDI during 2010–2016. CDI patients were divided into three groups according to CKD status: non-CKD (*n* = 363), CKD (*n* = 55) and those requiring dialysis (*n* = 63). Logistic regression analyses were performed to examine the association of CKD status with treatment failure. CDI patients receiving dialysis tended to have increased odds of metronidazole and overall treatment failure compared to non-CKD patients; adjusted odds ratios and 95% confidence intervals were 2.09 (1.03–4.21) and 2.18 (1.11–4.32) for metronidazole and overall treatment failure, respectively. However, CKD patients did not have increased odds of metronidazole or overall treatment failure compared to non-CKD patients, even where severe CDI was more prevalent in CKD patients. The incidence of symptomatic ileus or toxic megacolon did not differ among groups. Our results suggest that initial metronidazole therapy may be considered in CDI patients with non-dialysis CKD, but should not be considered in CDI patients undergoing dialysis.

## Introduction

*Clostridium difficile* infection (CDI) is the most common cause of transmissible nosocomial diarrhoea and is an increasingly frequent cause of morbidity and mortality among hospitalised patients [1, 2]. When the normal bacterial flora is disrupted, the colon is colonised with *C. difficile* bacteria, and released toxins can cause mucosal damage and inflammation [3]. CDI is strongly associated with antibiotic use [4], and other risk factors include older age, gastric acid suppression therapy, immunosuppression, prolonged hospitalisation and chronic kidney disease (CKD) [5–8].

The prevalence of patients with CKD or end-stage renal disease (ESRD) is increasing worldwide. Several studies have demonstrated that patients with CKD or ESRD have approximately two- to three-fold increased risk of CDI compared to those without CKD [5, 7, 9]. Impaired immune function, gastric acid suppression, increased antibiotic use and altered intestinal microbial flora in CKD patients all contribute to the development of CDI [7, 9, 10]. Outcomes of CDI in patients with CKD or ESRD are known to be worse than in those without CKD, and other contributory factors include increased mortality, longer hospital stays and higher costs associated with CDI [5, 6, 9, 11].

The commonly used antibiotics to treat CDI are oral metronidazole or oral vancomycin. Although Infectious Disease Society of America (IDSA) guidelines recently suggested either vancomycin or fidaxomicin for an initial episode of CDI due to a high prevalence of highly virulent strains in the United States, treatment guidance from the European Society of Clinical Microbiology and Infectious Diseases and Australasian Society for Infectious Disease continue to recommend metronidazole as first-line therapy of CDI in mild and moderate disease and vancomycin or fidaxomicin for severe disease, recurrent infection or for those with a high risk of recurrence [12, 13].

As the prevalence of hypervirulent strains is still low in Asia, and metronidazole resistance rates for *C. difficile* isolates have also been low, metronidazole may be cost-effective for initial therapy among patients with mild to moderate CDI in Asia [14, 15]. Oral metronidazole has been mainly used for initial therapy of CDI in CKD and ESRD patients in Korea, but the study for treatment failure in these patients was insufficient [9]. Therefore, we investigated the risk of CDI therapy treatment failure in patients who have non-dialysis CKD or ESRD.

## Methods

### Study population

This was a retrospective cohort study. Study populations were included in two secondary hospitals in Korea (Samsung Changwon and Changwon Fatima). Patients who were admitted to the hospital with a diagnosis of, and treatment for, an initial episode of CDI were included from June 2010 to November 2016. The diagnosis for CDI was confirmed by a stool toxin assay test or prominent endoscopic findings in patients with symptoms including persistent diarrhoea (± fever or abdominal pain). Estimated glomerular filtration rates (eGFR) were calculated according to the chronic kidney disease epidemiology collaboration equation. CKD was defined as eGFR <60 ml/min/1.73 m^2^ for more than 3 months. The study was approved by the Institutional Review Board of each hospital and exempt from informed consent.

### Data collection

All patient information was collected on age, sex, body mass index (BMI), comorbidities (hypertension and diabetes), use of proton pump inhibitor (PPI) or probiotics, history of previous antibiotics use within 30 days, continuous use of antibiotics during treatment of CDI, fever (body temperature >38.3 °C), shock, variation in white blood cells count (WBC), serum albumin, C-reactive protein (CRP) and serum creatinine. Initial treatment of CDI was discontinuation of other antibiotics and the use of oral metronidazole, oral vancomycin, alone or in combination. The 30-day mortality from the onset of CDI and/or its recurrence within two months from hospital discharge was recorded. The use of concomitant antibiotics was further categorised according to their CDI risk as high (carbapenem, 2nd-, 3rd- or 4th-generation cephalosporin, fluoroquinolone, lincosamide, pivampicillin or temocillin), medium (penicillin, penicillin combination, 1st-generation cephalosporin, macrolide, monobactam or streptogramin) or low (all other systemic antibiotics) and no concomitant antibiotic use [16]. All patients were classified into three groups according to CKD status: non-CKD, CKD (eGFR <60 ml/min/1.73 m^2^ for more than three months) and if receiving dialysis or not. Metronidazole treatment failure was defined as addition of, or change to, oral vancomycin for persistent or worsening symptoms such as diarrhoea, fever or increased abdominal discomfort attributed to CDI after three days of initial oral metronidazole therapy [12, 13]. Overall treatment failure was defined as the presence of persistent or worsening symptoms such as diarrhoea, fever or increased abdominal discomfort attributed to CDI after three days of initial CDI treatment, or the addition of further treatment if the physician considered that the current treatment had failed. Acute renal dysfunction was defined as an increase in serum creatinine >50% above baseline and fulminant colitis as the development of hypotension or shock, ileus or toxic megacolon [17].

### Statistical analysis

Continuous variables are presented as mean ± standard deviation (s.d.) or median (interquartile range) as appropriate. Analysis of variance, Kruskal–Wallis test and *χ*^2^ test were used to analyse differences between patient groups as appropriate. Our primary outcome was metronidazole treatment failure and the secondary outcome was overall treatment failure. Logistic regression analysis was used to examine the association of CKD status with treatment failure upon adjustment for age, sex and factors based on *a priori* knowledge including serum albumin, fever, risk-stratified concomitant antibiotic use, use of glycopeptide, number of antibiotics prescribed (⩾2 or <2), CRP and leukocytosis (WBC > 15 000/μl) [9, 18, 19]. For sensitivity analysis, we additionally adjusted for a history of previous CDI within 8 weeks before diagnosis of current episode, history of using metronidazole to treat other infectious diseases within 4 weeks before diagnosis of CDI and the length of hospital stay from patient admission to diagnosis of CDI, given that these variables may affect the association of CKD status and outcomes. As the continuous use of antibiotics was reported to be a strong predictor of metronidazole treatment failure, we performed subgroup analysis for the primary outcome according to the continuous use of antibiotics and likelihood ratio testing by adding an interaction term between CKD status and the continuous use of antibiotics to the adjusted logistic regression model [18]. All analyses were carried out using STATA version 14.2 (StataCorp LP, College Station, TX, USA).

## Results

### Patient characteristics

A total of 500 patients diagnosed with CDI was identified during the study period. After excluding 19 patients without data on treatment failure, 481 were finally included in this study. The proportion of CDI patients diagnosed with a stool toxin assay, endoscopic findings or both were 412 (85.7%), 23 (4.8%) and 46 (9.6%), respectively. Of them, 363 (75.5%) were diagnosed as non-CKD and 55 (11.5%) as CKD. Sixty-three (13.0%) patients received dialysis therapy. Baseline characteristics among the three groups are shown in Table 1. Patients aged ⩾65 years comprised 67% of the total, and males accounted for 49%. A total of 380 patients were initially treated with oral metronidazole: 282 (78%), 42 (76%) and 56 (89%) in non-CKD, CKD and dialysis patients, respectively. CKD and dialysis patients were more likely to receive PPI therapy and concomitant use of antibiotics compared to patients without CKD.

**Table 1.** Baseline characteristics of 481 patients with CDI according to CKD status

Variables	Total	No CKD	Non-dialysis CKD	Dialysis	*P* value
*N* (%)	481	363 (75.5)	55 (11.5)	63 (13.0)	
Age ⩾65 years	320 (67)	233 (64)	45 (82)	42 (67)	0.24
Male, %	236 (49)	170 (47)	26 (47)	40 (63)	0.026
BMI, kg/m^2^	22.0 (19.0–24.0)	21.0 (19.0–23.0)	21.0 (19.0–24.0)	23.0 (20.0–25.0)	0.028
Comorbidities, %					
Diabetes	129 (27)	75 (21)	21 (38)	33 (52)	<0.001
HTN	236 (49)	155 (43)	34 (62)	47 (78)	<0.001
Initial treatment of CDI, %					
Metronidazole	380 (79)	282 (78)	42 (76)	56 (89)	0.081
Vancomycin ± metronidazole	52 (11)	45 (12)	6 (11)	1 (2)	0.016
Discontinuation of antibiotics	49 (10)	36 (10)	7 (13)	6 (10)	0.905
Medication, %					
PPI	136 (29)	89 (25)	22 (40)	25 (40)	0.004
Probiotics	79 (16)	56 (15)	12 (22)	11 (17)	0.484
Previous antibiotics	403 (84)	303 (83)	45 (82)	55 (87)	0.557
Continuous use of antibiotics^a^	170 (35)	109 (30)	30 (55)	31 (49)	<0.001
Low-risk antibiotics for CDI	13 (8)	10 (9)	2 (7)	1 (3)	
Medium-risk antibiotics for CDI	41 (24)	26 (24)	7 (23)	8 (26)	
High-risk antibiotics for CDI	116 (68)	73 (67)	21 (70)	22 (71)	
Fever, BT >38.3	141 (29)	111 (31)	14 (25)	16 (25)	0.316
Shock, %	55 (11)	36 (10)	8 (15)	11 (17)	0.059
Fulminant colitis, %	74 (15)	47 (13)	12 (22)	15 (24)	0.03
Laboratory variables					
WBC, cells/μl	12 425±8171	12 093±8439	12 815±6513	14 002±7799	0.081
WBC > 15 000/μl	129 (27)	93 (26)	18 (33)	18 (29)	0.426
Serum albumin	2.9 ± 6.7	3.1 ± 7.7	2.4 ± 0.6	2.1 ± 0.5	0.209
CRP	50 (18–104)	47 (16–94)	60 (29–135)	66 (20–157)	0.004
Serum creatinine, mg/dl	0.8 (0.5–1.4)	0.7 (0.5–0.8)	1.6 (1.3–2.8)	5.1 (3.3–6.6)	<0.001
eGFR, ml/min/1.73 m^2^	89 (41–120)	103 (82–136)	35 (17–46)	10 (7–16)	<0.001

BMI, body mass index; BT, body temperature; HTN, hypertension; eGFR, estimated glomerular filtration rate; PPI, proton-pump inhibitor; WBC, white blood cells.

a High-risk antibiotics include carbapenem, 2nd-, 3rd- or 4th-generation cephalosporin, fluoroquinolone, lincosamide, pivampicillin or temocillin; medium-risk antibiotics include penicillin, penicillin combination, 1st-generation cephalosporin, macrolide, monobactam or streptogramin; low-risk antibiotics include all other systemic antibiotics.

### Metronidazole failure according to CKD status in CDI patients

Of the 380 patients who initially received metronidazole for CDI, the incidence of treatment failure was 20.3%; 18.8%, 16.7% and 30.4% in non-CKD, CKD and ESRD patients, respectively (*P* = 0.120). Dialysis patients with CDI tended to have increased odds of treatment failure for metronidazole compared to non-CKD patients (adjusted odds ratio (OR) 2.09, 95% confidence interval (CI) 1.03–4.21; *P* = 0.04). In subgroup analysis according to the concomitant use of antibiotics, there was a difference in association between CKD status and metronidazole failure. In patients not receiving antibiotics, dialysis was associated with an increased odds of metronidazole failure compared to non-CKD status (adjusted OR 2.87, 95% CIs 1.03–8.02; *P* = 0.044). On the other hand, in patients with the concomitant use of antibiotics, dialysis did not significantly increase the odds of metronidazole failure compared to the non-CKD status (Table 2). In test for interaction, however, it was not statistically significant (*P*_Interaction_ = 0.53). CKD patients did not have increased odds of metronidazole failure compared to non-CKD patients in overall and subgroup analyses. Likewise, in the sensitivity analysis, further adjusted for history of previous CDI, history of using metronidazole and the length of hospital stay before diagnosis of CDI, the results remained consistent (Table 3).

**Table 2.** Adjusted odds ratio for metronidazole treatment failure stratified by the continuous use of antibiotics among patients with CDI

				Use of concomitant antibiotics					
	Overall			Without			With		
Groups	OR^a^	95% CI	*P* value	OR^a^	95% CI	*P* value	OR^a^	95% CI	*P* value
Non-CKD	Reference			Reference			Reference		
Non-dialysis CKD	0.69	0.27–1.77	0.441	0.42	0.05–3.50	0.425	0.85	0.27–2.71	0.787
Dialysis	2.09	1.03–4.21	0.040	2.87	1.03–8.02	0.044	1.38	0.49–3.91	0.541

CKD, chronic kidney disease; CI, confidence interval; OR, odds ratio.

a OR was adjusted for age, sex, serum albumin, fever, risk-stratified concomitant antibiotic use, use of glycopeptide, number of antibiotics used (⩾2 or <2) and leukocytosis (WBC > 15 000/μl).

**Table 3.** Sensitivity analysis with further adjustment for treatment failure among patients with CDI

	Overall failure			Metronidazole failure		
Groups	OR^a^	95% CI	*P* value	OR^a^	95% CI	*P* value
Non-CKD	Reference			Reference		
Non-dialysis CKD	0.92	0.39–2.18	0.85	0.75	0.29–1.95	0.56
Dialysis	2.28	1.14–4.58	0.02	2.15	1.05–4.38	0.035

CKD, chronic kidney disease; CI, confidence interval; OR, odds ratio.

a OR was adjusted for age, sex, serum albumin, fever, risk-stratified concomitant antibiotic use, use of glycopeptide, number of antibiotics used (⩾2 or <2), leukocytosis (WBC > 15 000/μl), history of previous CDI within 8 weeks before diagnosis of CDI, history of using metronidazole within 4 weeks before diagnosis of CDI and length of hospital stay from patient admission to diagnosis of CDI.

### Overall treatment failure according to CKD status in CDI patients

Of 481 patients with CDI, the overall treatment failure was 16.6%. Compared to non-CKD patients with CDI, dialysis patients with CDI had increased odds of overall treatment failure; fully adjusted OR and 95% CI were 2.18 (1.11–4.32; *P* = 0.024). However, non-dialysis CKD was not associated with increased odds for overall treatment failure (adjusted OR 0.81, 95% CI 0.34–1.90; *P* = 0.623).

### Other outcomes according to CKD status in CDI patients

The incidence rate of acute renal dysfunction during CDI was higher in the non-dialysis CKD group compared to the non-CKD group (40% *vs.* 7.7%; *P* < 0.001). Fulminant colitis also showed an increase according to CKD status; 47 (13.0%), 12 (21.8%) and 15 (23.8%) in non-CKD, non-dialysis CKD and dialysis groups, respectively (*P* = 0.03). Among 457 patients with follow-up data, there was a significant difference in 30-day mortality according to CKD status with rates of 6.4%, 15.4% and 27.1% in the non-CKD, CKD and dialysis groups, respectively (*P* < 0.001). The rate of recurrent CDI did not differ among groups; 16.0%, 24.4% and 11.3% in non-CKD, CKD and dialysis groups, respectively (*P* = 0.204), and likewise for symptomatic ileus or toxic megacolon; 5.0%, 9.1% and 9.5% in non-CKD, CKD and dialysis groups, respectively (*P* = 0.223).

## Discussion

The present study has demonstrated that metronidazole treatment failure for CDI was higher in dialysis patients compared to those without CKD, especially in settings where antibiotics were not used concomitantly. Overall treatment failure for CDI was also significantly higher in dialysis patients compared to those without CKD. However, non-dialysis CKD patients did not have increased odds for treatment failure even when the presence of acute renal dysfunction, suggested as one of the signs of severe colitis, was more prevalent in CKD patients. The 30-day mortality rate incrementally increased according to CKD status (non-CKD, CKD and dialysis) with dialysis patients having the highest mortality. However, recurrent or complicated CDI accompanied by ileus or toxic megacolon did not differ according to CKD status.

Given that the virulent and epidemic ribotype 027 strain of *C. difficile* is one of the most commonly identified strains in the US and is associated with severity and mortality, a recent IDSA guideline has suggested that either vancomycin or fidaxomicin be used for an initial episode of CDI and metronidazole considered where access to vancomycin or fidaxomicin is limited [17]. This 027 strain produces a 16-fold higher concentration of toxin A and 23-fold higher concentration of toxin B, as well as the binary (transferase) toxin that leads to increased clostridial adherence to gut tissues [20, 21]. The associated mortality rate of the 027 strain is considered to be three-fold higher than for less virulent strains, and accounted for 28–50% of CDI in the US [20].

Until recently, oral metronidazole has been commonly used for the initial treatment for non-severe CDI in CKD patients, as well more widely in the general Korean population [9, 22]. In Korea, the 027 strain is still not common despite its first isolation in 2009 [23] and in 2011 accounted for only seven of 1251 isolates of *C. difficile* in Korea [24]. Similar results have been reported in Asia, including Japan [14, 15]. Furthermore, antimicrobial resistance appears to vary according to the geographical area with reported rates of metronidazole resistance of 15.6% in China from 2012 to 2015, 18.3% in Israel, 5.3% in Iran, 0.11% in Europe and 13.3% in the US (Texas) from 2007 to 2011 [25]. Vancomycin resistance is also variable globally, with rates of 0.87–2.28% of strains exhibiting intermediate resistance to vancomycin in Europe [26] compared with 17.9% resistance in a US-based national sentinel surveillance study [27]. In Korea, two recent studies have recorded full susceptibility to metronidazole and vancomycin of all isolates tested [21, 28] but in terms of the clinical response to treatment, the rate of metronidazole resistance was found to be 15.2% [9]. Patients with CKD and ESRD may have not only an increased risk of CDI, but also a higher risk of death compared to those without CKD [7, 29]. However, the significance of treatment failures for the most commonly used oral metronidazole among CKD and ESRD patients in Korea remains unclear. In this study, we did not observe increased odds of metronidazole treatment failure among CKD patients compared to non-CKD patients, even if severe CDI was more prevalent in CKD patients. In overall cohort, 20.3% of the patients experienced initial metronidazole treatment failure, but the failure rate in CKD patients was 16.7%, which was not higher than that in control patients. The clinical features of complicated CDI, such as ileus or toxic megacolon in CKD patients, were also not more frequent than in non-CKD patients, and the incidence of recurrent CDI was not significantly different among groups unlike the previous findings [30, 31]. Nevertheless, when compared with CDI patients without CKD, initial metronidazole therapy in those undergoing dialysis tended to be associated with an increased risk of treatment failure. As reported by others, concomitant antibiotic use alone in CDI patients was a strong predictor for metronidazole treatment failure [18]. Here, even if the association between dialysis and metronidazole failure was more pronounced in CDI patients without the continuous use of antibiotics, a statistically significant interaction effect was not demonstrated in our patient cohort.

Our study has some limitations. First, although we adjusted for potential confounding factors to investigate the association of CKD status with metronidazole treatment failure in CDI patients, confounding factors may have remained due to the observational study design. Moreover, as our study analysed data acquired retrospectively, incomplete or missing data in the medical records might have resulted in measurement bias or misclassification of outcomes. Second, because of the geographical differences in the distribution of epidemiologically dominant strain types and antimicrobial resistance, our findings may be limited to other countries with similar strain prevalence and antimicrobial resistance to Korea. Third, although elevated diagnostic parameters (e.g. WBC > 15 000 cells/μl, serum creatinine ⩾1.5 mg/dl for non-CKD patients or serum creatinine ⩾1.5 times the premorbid level for CKD patients) were used to differentiate CDI severity [17], there is no consensus regarding a definition of severe CDI, or the most important clinical indicators that should be used to differentiate severity. Further validation of these criteria is therefore warranted and also modified accordingly for patients with CKD or ESRD. Likewise, the role of acute renal dysfunction as a measure of CDI severity remains to be evaluated and validated in international studies [32, 33]. Lastly, we considered refractory CDI or treatment failure when CDI patients showed worsening symptoms or did not show clinical improvement after 3 days of initial therapy [12, 13]. In retrospect, this timing to determine successful outcomes may have led to the possibility of overestimating the incidence of metronidazole failure.

In conclusion, when dialysis patients were initially treated with oral metronidazole as a CDI treatment, they tended to have higher odds of treatment failure than non-CKD patients but this relationship was not evident for non-CKD patients. Our results suggest that initial metronidazole therapy may be considered in CDI patients with non-dialysis CKD, but should not be considered for CDI patients undergoing dialysis.
